# Acupuncture Induces the Proliferation and Differentiation of Endogenous Neural Stem Cells in Rats with Traumatic Brain Injury

**DOI:** 10.1155/2016/2047412

**Published:** 2016-05-25

**Authors:** Shuting Jiang, Weihao Chen, Yimin Zhang, Yujuan Zhang, Ailian Chen, Qiufu Dai, Shujun Lin, Hanyu Lin

**Affiliations:** ^1^Medical College, Jinan University, 601 Huangpu West Avenue, Guangzhou, Guangdong 510632, China; ^2^Xiangzhou District People's Hospital, 178 Lanpu Road, Xiangzhou District, Zhuhai, Guangdong 519070, China

## Abstract

*Purpose*. To investigate whether acupuncture induced the proliferation and differentiation of endogenous neural stem cells (NSCs) in a rat model of traumatic brain injury (TBI).* Methods*. 104 Sprague-Dawley rats were randomly divided into normal, model, and acupuncture groups. Each group was subdivided into three-day (3 d), seven-day (7 d), and fourteen-day (14 d) groups. The rat TBI model was established using Feeney's freefall epidural impact method. The rats in the acupuncture group were treated at acupoints (Baihui, Shuigou, Fengfu, Yamen, and bilateral Hegu). The normal and model groups did not receive acupuncture. The establishment of the rat TBI model and the therapeutic effect of acupuncture were assessed using neurobehavioral scoring and hematoxylin-eosin staining. The proliferation and differentiation of NSCs in TBI rats were analyzed using immunofluorescence microscopy.* Results*. The levels of nestin-expressing cells and bromodeoxyuridine/glial fibrillary acidic protein- (BrdU/GFAP-) and BrdU/S100 calcium-binding protein B-positive and BrdU/microtubule-associated protein 2- and BrdU/galactocerebrosidase-positive cells were more significantly increased at various time points in the acupuncture group than in the model group (*P* < 0.01), except for a decreased level of BrdU/GFAP-positive cells at 7 d and 14 d.* Conclusion*. Acupuncture induced the proliferation and differentiation of NSCs, thereby promoting neural repair in the TBI rats.

## 1. Introduction

With rapid societal development and the accompanying increase in traffic accidents, the rate of traumatic brain injury (TBI) has been growing [1–3]. How to promote neural repair following TBI has long been an urgent and intractable problem for the medical community. With research-driven advances, using neural stem cells (NSCs) to treat TBI has become a focus of intensive research in China and other countries [4, 5]. Our preliminary studies showed that acupuncture can promote the expression of nerve growth factors and brain-derived neurotrophic factors, as well as the expression of nestin and the production of astrocytes in the brain tissues of a rat model of TBI, thereby facilitating neural repair [6–9]. To further elucidate the effect of acupuncture, we used a rat model of TBI to assess the acupuncture-induced changes in relevant protein expression in NSCs at different time points after TBI. In addition, we assessed whether acupuncture induced NSC proliferation and differentiation after TBI.

## 2. Materials and Methods

### 2.1. Ethics Statement

This study was prospectively approved by the Laboratory Animal Ethics Committee of Jinan University (reference number 20150114205420) and was conducted in line with the Statute on the Administration of Laboratory Animal approved by China's Council 1988. All surgery was performed under chloral hydrate anesthesia, and all efforts were made to minimize suffering.

### 2.2. Reagents

The reagents used in this study included a hematoxylin-eosin (HE) staining kit (Solarbio, Beijing, China); a mouse anti-nestin antibody (Chemicon International Inc., Temecula, CA, USA); a mouse anti-bromodeoxyuridine (BrdU) antibody, a rabbit anti-glial fibrillary acidic protein (GFAP) antibody, a rabbit anti-microtubule-associated protein-2 (MAP-2) antibody, a rabbit anti-S100 calcium-binding protein B (S100B) antibody, and a rabbit anti-galactocerebroside (Galc) antibody (Cell Signaling, Beverly, MA, USA); a conjugated goat anti-mouse immunoglobulin G (IgG) (MultiSciences, Hangzhou, China); a conjugated goat anti-rabbit IgG (Cell Signaling, Beverly, MA, USA); and BrdU (Sigma-Aldrich, St. Louis, MO, USA).

### 2.3. Animals and Treatments

A total of 104 male Sprague-Dawley (SD) specific-pathogen-free (SPF) rats, each weighing 280 ± 20 g, were provided by the Guangdong Medical Experimental Animal Center (Guangzhou, China) under Animal Certificate Numbers 4400720000621 and 44007200017130. These animals were raised in the Experimental Animal Management Center of Jinan University (Guangzhou, China). The study was performed in the Medical Laboratory of Jinan University. The TBI model was established as follows: after 1 week of adaptive feeding, the 104 SPF-grade male SD rats were randomly divided into normal (A), model (B), and acupuncture (C) groups. Each group was then subdivided into 3 d, 7 d, and 14 d groups, in accordance with the chosen sampling and treatment times. The rats were weighed and were then anesthetized via an intraperitoneal injection of 10% chloral hydrate (0.3 mL/100 g). According to Feeney's freefall impact method, the skin of the skull was cut open, and the skull was drilled to open a window at 2 mm to the left of the sagittal suture and 1 mm posterior to the coronal suture, while the dura was kept intact. For groups B and C, a 20 g hammer was allowed to freely fall from a height of 30 cm. In this way, an impact force of ~60 g·cm caused local cerebral contusion and laceration of the left parietal lobe. The scalp was then sutured, and the rats were placed in cages under the routine conditions. The rats in group A were not treated. After establishment of the TBI model, the rats in group C were treated with acupuncture within 24 h, which was performed at a fixed time once a day at specific acupoints (Baihui, Shuigou, Fengfu, Yamen, and bilateral Hegu). A needle was inserted into the acupoints to a depth of 2 mm and was manipulated using the twirling-reducing method for 1 min for each acupoint. The needle was retained for a fixed period of 15 min, and acupuncture was performed once every 5 min. Although the rats in the other two groups (A and B) did not receive acupuncture, each of them was placed in a mouse fixator for 15 min to ensure that they experienced the conditions.

### 2.4. Instruments

The instruments used in this study included a water-jacketed thermostatic incubator (Fuma, Shanghai, China); electric thermostatic water bath (Boxun, Shanghai, China); paraffin-sectioning microtome, thermostatic water bath-slide drier, cryomicrotome, upright fluorescence microscope, and Qwin Plus image analysis system (Leica, Shanghai, China); and disposable number 26 0.5-inch Hwato acupuncture needles (Huatuo Medical Instruments, Suzhou, China).

### 2.5. Neurobehavioral Scoring

The base value of the neurobehavioral scores was determined using the modified neurological severity score (mNSS) method. The neurological behavior of rats was assessed at 1, 3, 7, and 14 d after the establishment of the model. The animals were scored for movement, sensation, and reflex. Rats with an mNSS score of 7–12 points were defined as moderate TBI models and were included in the experiment.

### 2.6. Sampling and Processing

Two days before sampling, the rats in all of the groups were intraperitoneally injected twice daily (8-hour interval) with BrdU (100 mg/kg body weight) dissolved in a saline solution. The rats were weighed and were then anesthetized via an intraperitoneal injection of 10% chloral hydrate (0.3 mL/100 g). The chest was cut open to expose the heart, and a number 12 perfusion needle was inserted into the aorta through the left ventricle under direct vision. The right atrial appendage was cut open, and rapid perfusion with 100 mL of normal saline was performed for 8 min, followed by rapid perfusion with 4% paraformaldehyde for ~15 min and then slow perfusion for 1 h. The rats were then decapitated, and brain tissue specimens were collected and fixed in 4% formaldehyde at room temperature. The specimens were dehydrated, embedded in paraffin or in optimal cutting temperature compound, and sectioned into 4 to 10 *μ*m-thick slices following conventional procedures.

### 2.7. Hematoxylin-Eosin (HE) Staining

HE staining was performed using an HE staining kit (Solarbio, Beijing, China) according to the manufacturer's instructions.

### 2.8. Immunofluorescence Assay

Frozen section specimens of brain tissue were washed with 0.01 M phosphate-buffered saline and then incubated in 50% formamide/2x SSC (0.3 M NaCl, 30 mM Na citrate) in a 65°C water bath for 2 h. After washing with 2x SSC, the specimens were treated with 2 N HCl at 37°C for 30 min, followed by treatment with pH 8.5 borate buffer at room temperature for 10 min. After being washed with 0.01 M PBS, the specimens were treated with PBS containing Tween 20 (PBST) for 15 min at room temperature and then blocked for 1 h in blocking buffer (5% goat serum, 0.1% bovine serum albumin, and 0.1% Triton X-100). Thereafter, the specimens were incubated with each of the primary antibody mixtures (nestin, 1 : 200; BrdU/GFAP, 1 : 200; BrdU/MAP-2, 1 : 200; BrdU/S100B, 1 : 200; and BrdU/Galc, 1 : 200) at 4°C for 36 h. After being washed with 0.01 M PBS, the specimens were incubated with a secondary antibody solution (conjugated goat anti-mouse IgG and/or goat anti-rabbit IgG) and in the dark inside a cassette at 37°C for 2 h. The specimens were then washed with 0.01 M PBS and mounted using 50% glycerol and then were observed and photographed under a fluorescence microscope. The number of doubly positive cells in the brain tissue on the injured side in each group was counted at different time points using Leica QWin image analysis software.

### 2.9. Statistical Analysis

Statistical analysis was performed using SPSS 13.0 statistical software (SPSS Inc., Chicago, IL, USA). The measurement data are represented as the mean values ± standard deviation ($\overset{-}{x}\pm S$). The experimental results were evaluated using two-factor two-level factorial analysis and analyses of variance (ANOVAs). The between-group arithmetic mean values were compared using the least significant difference (LSD) method. A *P* value of less than 0.05 was considered to indicate statistical significance.

## 3. Results

### 3.1. Neurobehavioral Scores

After the establishment of the model, groups B and C showed significantly increased mNSS scores compared with that of group A at 1 and 3 d (*P* < 0.01); there was no significant difference between the scores of groups B and C (*P* > 0.05). The mNSS scores of both groups B and C had declined at 7 d, with the decrease being more significant in group C than in group B; there was a significant difference between the scores of these two groups (*P* < 0.01). The mNSS score of group C had further decreased relative to that of group B at 14 d; however, the difference between the scores was not significant (*P* > 0.05) (Table 1).

### 3.2. HE Staining

**Figure 1:**
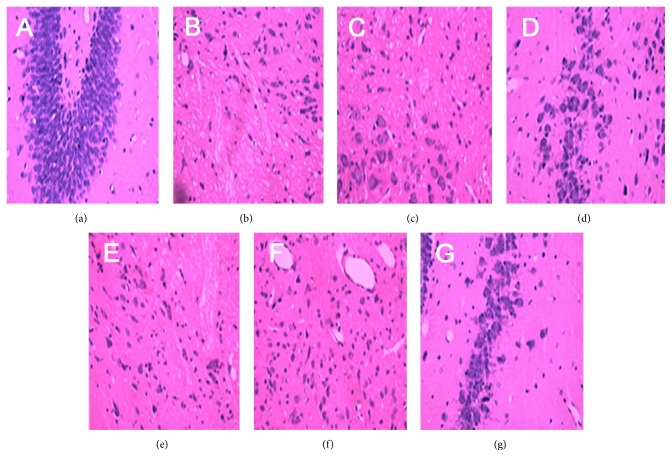
HE staining of rat brain tissue after TBI (×200). (a) Normal. (b) Model (3 d). (c) Model (7 d). (d) Model (14 d). (e) Acupuncture (3 d). (f) Acupuncture (7 d). (g) Acupuncture (14 d).

Microscopic observation revealed brain tissue with a clear, dense structure as well as neurons and glial cells with normal structures in group A at various time points. At 3 d, groups B and C showed loosely structured brain tissue, swollen neural and glial cells that had extensive vacuolar changes, and nuclei that were shifted and pyknotic; the cells were disarranged and a large amount of inflammatory cell infiltration was observed. At 7 and 14 d, cells in the brain tissues of groups B and C were pyknotic and karyolytic and the neurons were necrotic; glial cells were observed to have gradually emerged, and proliferative fibrous tissue had filled the injured site. Group B showed more significant pathological changes and a slower recovery compared with those of group C (Figure 1).

### 3.3. Immunofluorescence Staining

**Figure 2:**
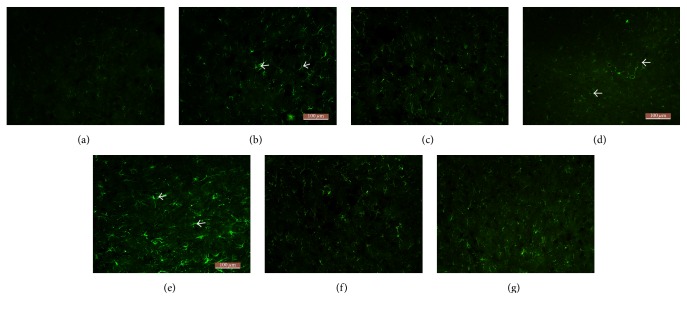
Nestin immunofluorescence staining of cells in rat brain tissue after traumatic brain injury (×200). (a) Normal. (b) Model (3 d). (c) Model (7 d). (d) Model (14 d). (e) Acupuncture (3 d). (f) Acupuncture (7 d). (g) Acupuncture (14 d).

Light microscopy revealed different numbers of nestin-stained cells in groups A, B, and C at 3, 7, and 14 d after the establishment of the TBI model. A large number of these cells appeared to contain green particles, mainly at the cell membrane and/or in the cytoplasm. Higher levels of nestin staining were observed in groups B and C at different time points. In particular, the number of nestin-positive cells was markedly increased in group C compared with that in group B at different time points (*P* < 0.01) (Table 2 and Figure 2).

**Figure 3:**
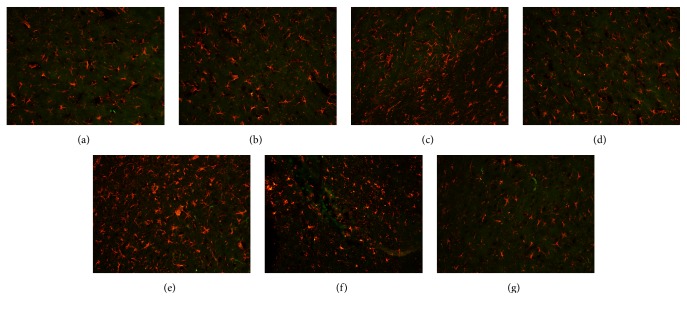
BrdU/GFAP double-positive cells in rat brain tissue after traumatic brain injury (×200). (a) Normal. (b) Model (3 d). (c) Model (7 d). (d) Model (14 d). (e) Acupuncture (3 d). (f) Acupuncture (7 d). (g) Acupuncture (14 d).

A significant difference between the levels of BrdU/GFAP double-stained cells in groups A and B was observed at each time point (*P* < 0.01). Compared with the number of these cells in group A, the number in group B was significantly increased at 3 d; the increase was more obvious at 7 d, and the number of these cells remained at a high level at 14 d. Moreover, significant differences in the numbers of these cells in groups B and C were observed at different time points (*P* < 0.01 and *P* < 0.05). The number in group C was significantly more increased than that in group B at 3 d and then markedly decreased to a lower level than that in group B at 7 d; this number was close to normal at 14 d (Table 3 and Figure 3).

**Figure 4:**
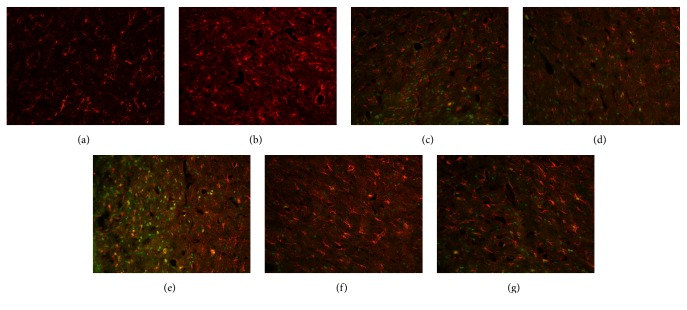
BrdU/S100B double-positive cells in rat brain tissue after traumatic brain injury (×200). (a) Normal. (b) Model (3 d). (c) Model (7 d). (d) Model (14 d). (e) Acupuncture (3 d). (f) Acupuncture (7 d). (g) Acupuncture (14 d).

Comparison between groups A and B revealed a significant difference in the levels of BrdU/S100B double-stained cells at each time point. Compared with the number of these cells in group A, the number in group B was increased at 3 d (*P* < 0.01) and markedly decreased at 7 d and had generally returned to normal at 14 d (*P* < 0.05). A significant difference between the numbers of these cells in groups B and C was also observed at each time point (*P* < 0.01). Compared with the number of these cells in group B, that in group C was significantly increased at 3 d, had gradually decreased by 7 d, and remained high at 14 d (Table 4 and Figure 4).

**Figure 5:**
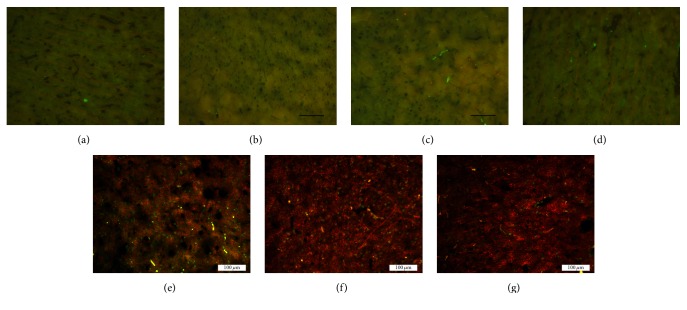
BrdU/MAP-2 double-positive cells in rat brain tissue after traumatic brain injury (×200). (a) Normal. (b) Model (3 d). (c) Model (7 d). (d) Model (14 d). (e) Acupuncture (3 d). (f) Acupuncture (7 d). (g) Acupuncture (14 d).

There was a significant difference between the levels of BrdU/MAP-2 double-stained cells in groups A and B at each time point (*P* < 0.01). Compared with the number of these cells in group A, the number in group B was significantly increased at 3 d, and this increase was larger at 7 d; however, a downward trend was observed at 14 d. The numbers of these cells in groups B and C were also significantly different at each time point (*P* < 0.01). Compared with the number of these cells in group B, that in group C was increased at 3 d and significantly increased at 7 d, and the number of these cells remained high at 14 d (Table 5 and Figure 5).

**Figure 6:**
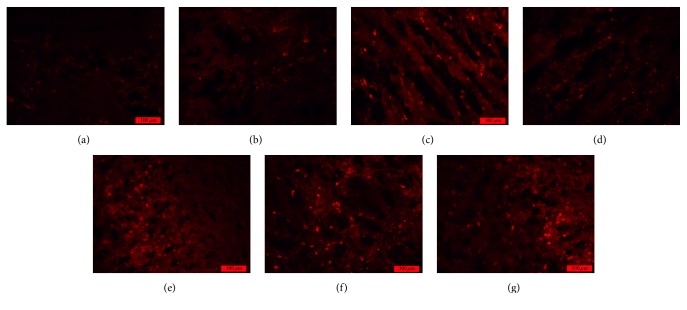
BrdU/Galc double-positive cells in rat brain tissue after traumatic brain injury (×200). (a) Normal. (b) Model (3 d). (c) Model (7 d). (d) Model (14 d). (e) Acupuncture (3 d). (f) Acupuncture (7 d). (g) Acupuncture (14 d).

Groups A and B showed a significant difference in the levels of BrdU/Galc double-stained cells at each time point (*P* < 0.01). Compared with the number of these cells in group A, the number in group B exhibited an upward trend at 3, 7, and 14 d. A significant difference between the number of these cells in groups B and C was also observed at each time point (*P* < 0.01). Compared with the number of these cells in group B, the number in group C was more significantly increased at 3, 7, and 14 d (Table 6 and Figure 6).

## 4. Discussion

Nestin is presently recognized as an early major specific marker of NSCs and/or progenitor cells. Nestin-positive cells are typically regarded as stem cells with a differentiation potential in an initial state. Nestin expression ceases when NSCs differentiate into neurons, astrocytes, or oligodendrocytes. Studies [10–12] have revealed no endogenous presence of BrdU, an analog of thymidine, in tissues and cells; after the* in vivo* injection of BrdU into TBI rats, cells incorporate this compound into newly synthesized DNA, and thus BrdU-positive cells are regarded as cells with proliferative activity. Therefore, BrdU is a more reliable marker for tracking the* in situ* mobilization of NSCs. In the present study, we observed changes in the proliferation and differentiation of renewing cells using immunofluorescence double staining with a monoclonal anti-BrdU antibody and antibodies directed against other NSC-specific proteins. However, because the production of the original anti-BrdU antibody was suspended, we were unable to complete the proposed nestin/BrdU double staining. Therefore, we could perform only single antinestin staining.

GFAP is a specific marker protein of mature astrocytes; it is also a specific marker protein of NSCs after TBI. In the central nervous system, S100B is a sugar-free, fat-free, and phosphorous-free acidic calcium-binding protein [13]. This protein is primarily located in astrocytes and oligodendrocytes in the central nervous system, as well as in Schwann cells in the peripheral nervous system. S100B is a specific marker protein of nerve injury produced mainly by astrocytes. MAP-2 is a cytoskeletal component of neurons that plays a role in the growth of neurons and in their repair after injury [14, 15]. This protein is mainly distributed in the dendrites of neurons and with the microtubules within the cell body and is a specific marker for the early injury of neurons. Oligodendrocytes are myelin-forming cells in the central nervous system that account for the lowest proportion of the natural products of NSC differentiation [16]. Galc is a specific marker of oligodendrocytes in the nervous system. Yu et al. [17] reported that a combination of PDGF and BFGF promotes the directional differentiation of oligodendrocytes; these two factors play an essential role in regulating oligodendrocyte differentiation. This result is in agreement with those of our previous studies [8, 18], which demonstrated that acupuncture can promote EGF and BFGF expression.

In this study, we observed significantly higher numbers of nestin- and BrdU/S100B-positive cells in the acupuncture group compared with those in the model group at different time points after TBI. The number of BrdU-positive/GFAP-expressing cells in the acupuncture group was significantly higher than that in the model group at 3 d, and this number then decreased to a level lower than that in the model group at 7 d. The number of these cells in the acupuncture group was close to that in the normal group at 14 d, whereas the number in the model group was still maintained at a higher level. It has been reported that astrocytes are most closely associated with the formation of glial scars [19]. After TBI, inflammation occurs in the local nerves of the brain, which activates microglia and astrocytes. Injured or dead cells and tissues are devoured and cleared through immune processes, and the injured lesion site is enwrapped via glial cell hypertrophy and proliferation to form a glial scar. After their activation, astrocytes have a positive effect on neural repair during different periods of differentiation. However, mature astrocytes can secrete harmful factors to form a chemical glial barrier (glial scar), which seriously affects nerve regeneration and prevents axonal elongation. Wu et al. [20] have reported that it is difficult to control glial cell proliferation and glial scar formation using a single approach. Our results showed that acupuncture can not only promote astrocyte proliferation and differentiation but also inhibit their excessive proliferation and differentiation. MAP-2 was gradually lost in the TBI site, indicating cytoskeletal degradation in the injured neuronal cells. This process could result in delayed neuronal death and might be associated with the calcium-mediated activation of some proteases after TBI. Appropriate acupuncture intervention after injury may ameliorate cytoskeletal degradation, maintain the shapes of cells, and reduce the incidence of delayed neuronal death. Our results proved that, during the early post-TBI stage, acupuncture can promote the proliferation and differentiation of NSCs and glial cells, which is conducive to control neuronal necrosis. During the late stage, acupuncture can inhibit the excessive proliferation and differentiation of glial cells, which is conducive to the regeneration of neurons and oligodendrocytes as well as to improve the capacity for neural tissue repair. TBI also activated the production of a number of NSCs for neural regeneration and repair compared with the levels observed in the normal group; however, this effect was limited when compared with that observed in the acupuncture group. This study also has limitations and shortcomings. We did not design a sham acupuncture group control for group C and a group of normal animals which received acupuncture treatment because of limited funding. But we will design a more complete experiment in future research.

In summary, acupuncture induced the proliferation of endogenous NSCs and their differentiation toward neuronal and oligodendrocyte lineages. The treatment bidirectionally regulated astrocyte proliferation and differentiation, which had a therapeutic effect on neural regeneration and repair in TBI rats.

## Figures and Tables

**Table 1:** Comparison of the mNSS scores of different groups of rats with traumatic brain injury ($\overset{-}{x}\pm S$).

Group	Number of cases	1 d	3 d	7 d	14 d
A	32	1.32 ± 0.47	1.33 ± 0.35	1.19 ± 0.73	1.17 ± 0.76
B	36	8.27 ± 0.37^*∗*^	8.57 ± 0.80^*∗*^	7.56 ± 0.91^*∗*^	3.83 ± 1.30^*∗*^
C	36	8.93 ± 0.72^*∗*^	8.02 ± 1.07^*∗*^	5.42 ± 0.95^*∗*▲^	3.01 ± 0.54^*∗*△^

Compared with group A, *∗* indicates *P* < 0.01 and, compared with group B, ▲ indicates *P* < 0.01 and △ indicates *P* > 0.05.

**Table 2:** Numbers of nestin-positive cells in the brain tissue on the injured side in different groups of rats ($\overset{-}{x}\pm S$).

Group	Number of cases	3 d	7 d	14 d
A	9	8.00 ± 4.09	7.88 ± 3.54	7.11 ± 3.44
B	9	53.77 ± 7.12^*∗*^	43.00 ± 7.68^*∗*^	23.33 ± 4.50^*∗*^
C	9	74.11 ± 10.54^*∗*▲^	63.66 ± 11.83^*∗*▲^	41.00 ± 6.12^*∗*▲^

Compared with group A, *∗* indicates *P* < 0.01 and, compared with group B, ▲ indicates *P* < 0.01.

**Table 3:** Numbers of BrdU/GFAP double-positive cells in the brain tissue on the injured side in different groups of rats ($\overset{-}{x}\pm S$).

Group	Number of cases	3 d	7 d	14 d
A	9	5.30 ± 2.12	6.03 ± 2.82	6.18 ± 2.91
B	9	13.81 ± 4.74^*∗*^	15.67 ± 4.93^*∗*^	12.73 ± 4.32^*∗*^
C	9	26.77 ± 6.06^*∗*▲^	10.57 ± 2.98^*∗*△^	7.51 ± 2.06^*∗*▲^

Compared with group A, *∗* indicates *P* < 0.01 and, compared with group B, ▲ indicates *P* < 0.01 and △ indicates *P* < 0.05.

**Table 4:** Numbers of BrdU/S100B double-positive cells in the brain tissue on the injured side in different groups of rats ($\overset{-}{x}\pm S$).

Group	Number of cases	3 d	7 d	14 d
A	9	1.76 ± 0.83	1.61 ± 0.53	1.73 ± 0.59
B	9	8.37 ± 1.86^*∗*△^	3.97 ± 1.93^*∗*^	2.87 ± 1.07^*∗*^
C	9	17.37 ± 7.39^*∗*▲^	11.5 ± 2.99^*∗*▲^	5.40 ± 1.76^*∗*▲^

Compared with group A, △ indicates *P* < 0.01 and *∗* indicates *P* < 0.05 and, compared with group B, ▲ indicates *P* < 0.01.

**Table 5:** Numbers of BrdU/MAP-2 double-positive cells in the brain tissue on the injured side in different groups of rats ($\overset{-}{x}\pm S$).

Group	Number of cases	3 d	7 d	14 d
A	9	4.67 ± 1.27	3.54 ± 1.57	3.10 ± 1.89
B	9	8.06 ± 1.91^*∗*^	10.13 ± 3.88^*∗*^	7.63 ± 2.05^*∗*^
C	9	10.20 ± 3.09^*∗*▲^	18.70 ± 5.04^*∗*▲^	16.5 ± 1.78^*∗*▲^

Compared with group A, *∗* indicates *P* < 0.01 and, compared with group B, ▲ indicates *P* < 0.01.

**Table 6:** Numbers of BrdU/Galc double-positive cells in the brain tissue on the injured side in different groups of rats ($\overset{-}{x}\pm S$).

Group	Number of cases	3 d	7 d	14 d
A	9	0.63 ± 0.75	0.92 ± 0.63	0.50 ± 0.74
B	9	1.23 ± 0.97^*∗*^	1.90 ± 1.37^*∗*^	1.51 ± 0.85^*∗*^
C	9	2.32 ± 1.83^*∗*▲^	3.87 ± 1.66^*∗*▲^	3.16 ± 1.58^*∗*▲^

Compared with group A, *∗* indicates *P* < 0.01 and, compared with group B, ▲ indicates *P* < 0.01.
